# Increased medial meniscus extrusion on ultrasonography in highly competitive rugby players: A cross-sectional study

**DOI:** 10.1371/journal.pone.0354478

**Published:** 2026-07-23

**Authors:** Kaoru Okano, Norimitsu Kinoshita, Ayame Matsuoka, Hiroaki Seto

**Affiliations:** Graduate School of Sports and Health Studies, Hosei University, Machida, Tokyo, Japan; Monash University, AUSTRALIA

## Abstract

Medial meniscal extrusion (MME) has been shown to be associated with the onset and progression of knee osteoarthritis (KOA); however, evidence focusing on young athletes without a history of knee injury remains limited. This study aimed to determine whether MME is already increased in young competitive rugby players compared with controls. The participants comprised 94 male collegiate rugby players without a history of knee trauma or surgery (165 knees) and 42 male university students serving as controls (81 knees). MME was assessed bilaterally using ultrasonography (US) in three knee positions: supine, figure-4, and single-leg standing. Between-group comparisons were performed using linear mixed-effects models with group and side as fixed effects, body mass index as a covariate, and participant ID as a random effect. Estimated marginal means and corresponding 95% confidence intervals were derived from the models. *p*-values for the group effect were adjusted across knee positions using the Benjamini–Hochberg procedure. The rugby group exhibited significantly greater MME than the control group across all knee positions (all *p* < 0.001). Greater MME was observed in young rugby players even in the absence of clinically apparent knee injury. These findings suggest an association between participation in high-intensity rugby and increased MME, which may reflect early changes related to KOA. Early identification of these alterations provides an opportunity for timely intervention aimed at preserving long-term joint health in contact sport athletes. However, because this study was cross-sectional, causality cannot be inferred. US represents a practical method for evaluating meniscal behavior under weight-bearing conditions that reflect sport-specific loading characteristics, and future longitudinal and interventional studies are warranted to establish practical MME assessment approaches and effective KOA prevention strategies in rugby players.

## Introduction

Medial meniscal extrusion (MME) is a pathological condition in which the medial meniscus (MM) is displaced beyond the tibial articular margin toward the extra-articular direction. Numerous previous studies have identified MME as an important factor involved in both the onset and progression of knee osteoarthritis (KOA) [1–3]. Previous studies have reported that MME shows a significant correlation with the Kellgren–Lawrence grade [1], and that prospective cohort studies have demonstrated its ability to predict the development and progression of KOA over a five-year follow-up period [2]. Furthermore, even in early-stage KOA with Kellgren–Lawrence grades 0 or 1, MME has been shown to be associated with a reduction in medial tibial cartilage volume. Regardless of the presence of bone marrow lesions, the presence of MME itself has been reported to be associated with cartilage loss, KOA progression, and an increased risk of total knee arthroplasty [3]. Epidemiological studies [4–15] and biomechanical investigations [16–20] have demonstrated that, as MME increases, the load-distributing function of the meniscus within the knee joint is compromised. As a consequence, localized contact stress within the joint is increased, which is thought to promote cartilage damage and thereby contribute to the onset and progression of KOA [21,22]. Therefore, an increase in MME can be interpreted as an indicator that early pathological changes related to KOA may already be present.

In highly competitive rugby players, increases in MME may increase even at a young age and in the absence of an apparent history of knee joint injury. Rugby is a sport that involves high-speed running, repeated accelerations and decelerations, and frequent collision events [23]. Recent analyses using global positioning system technology have demonstrated associations between these external loads and neuromuscular fatigue [23], suggesting that rugby imposes high physical demands. Indeed, former elite rugby players have been reported to exhibit a 2.3-fold higher prevalence of KOA compared with former athletes from non-contact sports [24]. In addition, match-related injury incidence in both elite and amateur male rugby league has been reported to be high [25,26], even when compared with other high-load sports such as American football, soccer, and cricket [27–29]. Furthermore, a longitudinal study evaluating femoral articular cartilage thickness and cross-sectional area using ultrasonography (US) over two consecutive seasons in collegiate rugby players reported that continued participation in rugby was associated with significant reductions in these cartilage indices, regardless of a prior history of knee joint injury [30]. Collectively, these findings indicate that the knee joints of rugby players are subjected to greater and more repetitive mechanical loading, which, given the association between MME and an increased risk of cartilage damage [31], may contribute to increased MME. However, to the best of our knowledge, no studies have specifically examined MME in young rugby players with clinically healthy knees.

Traditionally, magnetic resonance imaging (MRI) has been regarded as the gold standard for the morphological evaluation of the meniscus, including MME [32,33]. While MRI enables detailed visualization of meniscal and cartilage morphology, several limitations have been noted, including susceptibility to involuntary patient motion, restriction to static joint assessment, and high examination costs [32,33]. In contrast, US, although not considered the gold standard, offers advantages such as the ability to assess the knee under weight-bearing conditions and to perform dynamic evaluations during knee flexion and extension, which are difficult to achieve with MRI [34,35]. MME is known to vary depending on weight-bearing status and knee positions [36,37], and the magnitude of these changes has been reported to differ according to the presence or absence of meniscal pathology [38,39]. Therefore, assessment under weight-bearing conditions is considered desirable to obtain clinically relevant and realistic measurements [40]. In a study of patients with chronic knee pain, semi-quantitative and quantitative US assessments of MME demonstrated moderate to substantial agreement with MRI findings obtained in the same imaging plane, and US showed high sensitivity and good specificity as an evaluative method [32]. Taken together, for athletes who are routinely exposed to mechanical loading on the knee joint in sporting environments, US-based evaluation of MME—including single-leg standing assessments—represents a rational approach that reflects sport-specific loading characteristics.

Therefore, the purpose of the present study was to determine whether increased MME is already present in young rugby players with clinically healthy knees by conducting between-group comparisons across populations with differing levels of competitive exposure. We hypothesized that young rugby players would exhibit significantly greater MME than general university students.

## Materials and methods

### Research ethics

This study was approved by the Research Ethics Committee of Hosei University in May 2023 and was conducted in accordance with the Declaration of Helsinki (approval number: 2023_02). Participant recruitment was conducted between April 1, 2024 and April 30, 2025. All participants were informed of the purpose, procedures, benefits, and potential risks of the study, as well as the handling of study data, and provided written informed consent prior to participation.

### Participants

Initially, 112 highly competitive male university rugby players (224 knees; rugby group) and 42 male university students (84 knees; control group) were recruited for this study.

The rugby players were members of a collegiate rugby team competing in the first division of the Kanto University Rugby League. As part of their competitive training, they engaged in approximately 2 hours of rugby practice five days per week and performed resistance training three to four times per week.

The control group consisted of university students majoring in sports and health studies who were not engaged in high-level competitive athletic training. At the time of assessment, some participants engaged in little regular physical activity, while among those who were physically active, the frequency was once to at most three times per week.

Knees with a physician-confirmed history of injury or surgery, as reported in a questionnaire, were excluded after MME measurement. This exclusion was applied to clarify the relationship between repetitive knee loading due to sports participation and MME by eliminating cases in which MME might have been elevated because of prior knee damage.

### Questionnaire survey

A custom questionnaire was distributed to participants prior to measurement. The questionnaire collected information on age, height, weight, previous knee injury, and history of knee surgery. In addition, years of rugby experience were recorded for participants in the rugby group.

### Experimental procedures

On the same day, MME of both knees was assessed using US in the following order: supine, figure-4, and single-leg standing. All measurements were conducted on days without rugby practice to minimize the acute effects of rugby-specific loading on MME.

MME was assessed at the level of the medial collateral ligament (MCL), defined by a reference line connecting the distal–medial cortex of the femur and the medial ridge of the tibial cortex.

### MME assessment

**Fig 1:**
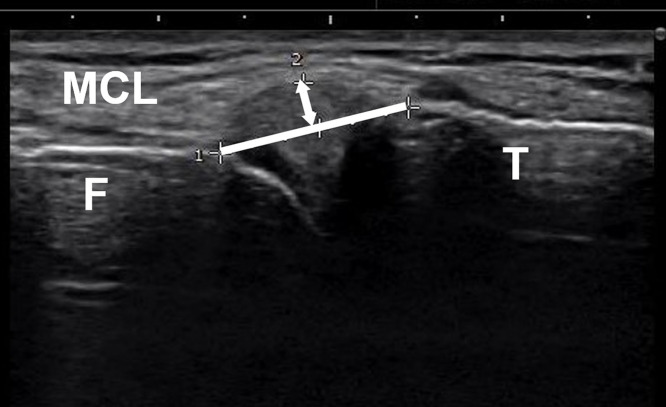
US measurement of medial meniscus extrusion MME. MME = medial meniscus extrusion. US = ultrasonography. F = femur. T = tibia. MCL = medial collateral ligament. ↕ = MME distance.

US measurements were performed using an US system (SONIMAGE HS1; Konica Minolta, Tokyo, Japan) with a linear probe (L18–4). MME was defined as the distance from the line connecting the femoral and tibial cortical bones to the medial edge of the medial meniscus, measured at the site where the MCL was most clearly visualized (Fig 1) [37]. When osteophytes were present, the cortical bone excluding osteophytes was used as the reference. A perpendicular measurement line was visually confirmed and manually drawn by the examiner. All MME measurements were performed by a single examiner (K.O.). The examiner was a graduate student with approximately four years of experience in musculoskeletal US.

### Measurement positions

The measurement procedures for each knee position are described below.

In the supine, measurements were performed with the knee fully extended, and a 4-kg medicine ball was placed and fixed on the lateral side of the foot to ensure that the patella was oriented vertically upward and to prevent external rotation of the lower limb. This position was selected based on previous studies evaluating MME under non–weight-bearing conditions [1,36,38].

For the figure-4, the knee flexion angle was set at 90°, in accordance with the method described by Hoshikawa et al. [38]. This position has been reported to be associated with increased MME in patients with medial meniscus posterior root tears [38], suggesting that it may impose mechanical loading particularly on the posterior root of the medial meniscus. For single-leg standing measurements, participants held a cylindrical weighted exercise tool (ViPR; Bravo Group Inc.) vertically while measurements were performed under single-leg weight-bearing conditions. A 4-kg ViPR was used to stabilize the trunk, reduce compensatory movements, and ensure a standardized measurement posture across participants. The knee joint was maintained in a fully extended position, and no participants exhibited hyperextension. Although previous studies have commonly evaluated MME under double-leg weight-bearing conditions, single-leg weight-bearing was adopted in the present study. This decision was based on the assumption that rugby-specific movements, such as acceleration, deceleration, and changes of direction, frequently involve single-leg loading; therefore, single-leg weight-bearing measurements were considered to better reflect the mechanical loading applied to the knee joint during actual rugby activities.

### Reliability

All measurements were conducted by a single examiner to ensure consistency. Intra-rater reproducibility was evaluated in a subsample of five university students with healthy knees, who were measured twice within one week in all positions. Intra-rater reliability exceeded 0.90 in all positions: 0.97 (95% confidence interval [CI]: 0.89–0.99) in the supine, 0.94 (95% CI: 0.78–0.98) in the figure-4, and 0.94 (95% CI: 0.77–0.98) in the single-leg standing.

### Statistical analyses

Statistical analyses were performed using SPSS software (version 29.0; IBM Corp., Armonk, NY, USA) and Microsoft Excel (version 2406; Microsoft Corporation, Redmond, WA, USA).

No a priori sample size calculation or power analysis was performed; instead, the sample size was determined based on the number of participants available during the study period. Continuous variables are presented as mean ± standard deviation (SD) and 95% confidence intervals (CI). Normality of the data was assessed using Q–Q plots. In the rugby group, two extreme outliers were identified and confirmed to correspond to knees with a history of surgery or injury; these cases were excluded from further analyses. Similarly, one knee in the control group that met the predefined exclusion criteria was identified during data verification and excluded prior to the final analyses. This knee was therefore removed prior to the final analyses. After these exclusions, no marked skewness or kurtosis was observed, and the data were considered approximately normally distributed.

Between-group comparisons at each knee position were conducted using separate linear mixed-effects models to account for the non-independence of bilateral knee data. Separate models were constructed for each knee position because the primary aim of this study was to evaluate between-group differences within each position. In these models, MME was included as the dependent variable, and group (rugby vs. control) and side (left vs. right) were treated as fixed effects. Body mass index (BMI) was included as a covariate based on prior evidence indicating an association between BMI and increased MME [1,6,36]. Participant ID was included as a random intercept to account for within-subject clustering of bilateral knees.

A variance components covariance structure was assumed, implying homogeneity of variance, and the models were estimated using restricted maximum likelihood. Estimated marginal means of MME and their 95% confidence intervals were calculated from the models and are reported for each group. Because between-group comparisons were performed across three knee positions, *p*-values were additionally adjusted for multiple comparisons using the Benjamini–Hochberg procedure to control the false discovery rate. Statistical significance was set at *p* < 0.05.

## Results

### Participant characteristics

**Fig 2:**
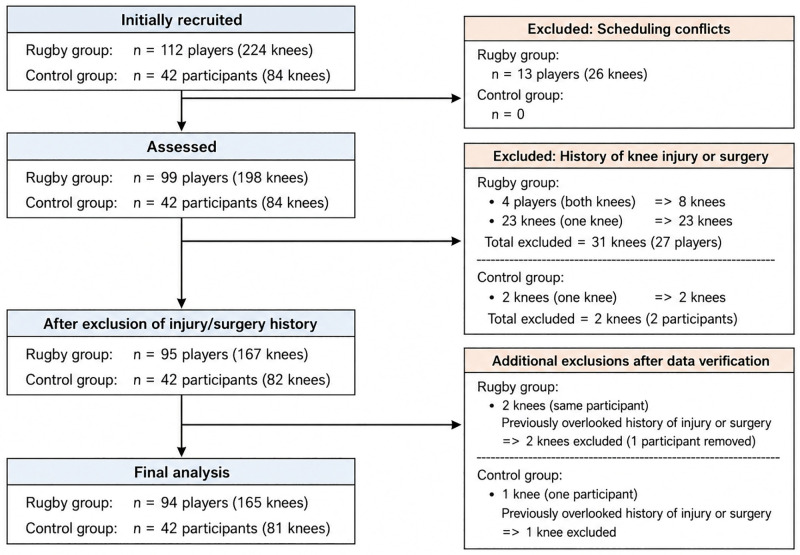
Flow diagram of participant selection and exclusion process.

The demographic characteristics of the participants included in the final analysis are presented in Table 1. The participant selection and exclusion process is illustrated in Fig 2.

**Table 1:** Participants’ demographic data.

Characteristic	Rugby group	Control group
**Knee**	165	81
**Age, years**	19.3 ± 1.0 (19.1-19.5)	18.8 ± 1.2 (18.4-19.2)
**Height, cm**	175.5 ± 5.5 (174.4-176.6)	169.9 ± 6.3 (167.9-171.8)
**Weight, kg**	91.0 ± 14.2 (88.1-93.9)	64.9 ± 10.4 (61.7-68.2)
**BMI, kg/m** ^**2**^	29.6 ± 4.1 (28.7-30.3)	21.9 ± 2.7 (21.5-22.3)
**Rugby experience, years**	11.1 ± 3.8 (10.4-12.0)	―

Notes: Values are presented as mean ± standard deviation with 95% CI. BMI= body mass index. CI= confidence intervals.

In the rugby group, 112 highly competitive university rugby players were initially recruited. Of these, 13 players were excluded because measurements could not be conducted on the scheduled data collection day due to scheduling conflicts. Among the remaining 99 players (198 knees) who were assessed, questionnaire responses revealed a history of knee injury or surgery in both knees in 4 players and in one knee in 23 players. These cases were excluded, leaving data from 95 players and 167 knees for analysis.

During the data cleaning process, two clear outliers were identified. A detailed re-examination of these cases revealed previously overlooked information regarding a history of knee injury or surgery. In accordance with the predefined exclusion criteria, these knees were also excluded from the analysis. Both outlier knees belonged to the same participant; therefore, the exclusion resulted in a reduction of one participant. Consequently, the final rugby group consisted of 94 players and 165 healthy knees.

In the control group, 42 university students majoring in sports and health studies (84 knees) were initially enrolled. After excluding knees with a history of injury or surgery, 42 participants and 82 knees remained. During verification of participant identifiers for the linear mixed-effects analyses, one additional knee was found to have a previously overlooked history of injury or surgery and was therefore excluded in accordance with the predefined exclusion criteria. Consequently, the final control group consisted of 42 participants and 81 healthy knees.

MME values for each knee position in both groups before statistical adjustment are presented in Table 2.

**Table 2:** MME by position in each group.

	Supine (mm)	Figure-4 (mm)	Single-leg standing (mm)
**Rugby group**	2.6 ± 0.6 (2.48-2.65)	1.4 ± 0.5 (1.28-1.44)	3.0 ± 0.6 (2.90-3.09)
**Control group**	2.2 ± 0.5 (2.12-2.35)	0.9 ± 0.5 (0.82-0.96)	2.5 ± 0.6 (2.45-2.63)

Notes: Values are presented as mean ± standard deviation with 95% CI. MME= medial meniscus extrusion. CI= confidence intervals.

Participants in both the rugby and control groups are shown in a single vertical flow. Exclusion criteria and reasons are presented on the right side. Notably, two outlier knees in the rugby group were derived from the same participant, resulting in the exclusion of one participant.

### Between-group comparisons of MME by knee position

The results of the between-group comparisons between the rugby and control groups are presented in Table 3. Using linear mixed-effects models, a significant group effect was observed across all knee positions, with rugby players demonstrating greater MME than controls, even after adjusting for side and BMI. No significant side effect was detected in any of the evaluated positions (all *p* > 0.05). BMI was not significantly associated with MME in the supine or figure-4 positions; however, a significant negative association was observed in the single-leg standing position (β = −0.033, 95% CI: −0.06 to −0.01, *p* = 0.01).

**Table 3:** Comparison of MME across different positions between rugby players and controls using a linear mixed-effects model.

Position	Variable	β (Estimate)	95% CI	*p*-value
**Supine**	Group	0.453	0.21 to 0.70	<0.001*
	Side	−0.052	−0.14 to 0.04	0.24
	BMI	−0.013	−0.04 to 0.01	0.24
**Figure-4**	Group	0.48	0.27 to 0.69	<0.001*
	Side	−0.095	−0.20 to 0.01	0.07
	BMI	0.002	−0.02 to 0.02	0.84
**Single-leg standing**	Group	0.703	0.44 to 0.97	<0.001*
	Side	−0.085	−0.18 to 0.01	0.08
	BMI	−0.033	−0.06 to −0.01	0.01*

Notes: Between-group comparisons were performed using a linear mixed-effects model with group and side as fixed effects, BMI as a covariate, and participant ID as a random effect to account for within-subject correlation. *p*-values for the group effect were adjusted across knee positions using the Benjamini–Hochberg procedure. *p < 0.05. MME = medial meniscus extrusion. CI = confidence intervals. BMI = body mass index.

**Fig 3:**
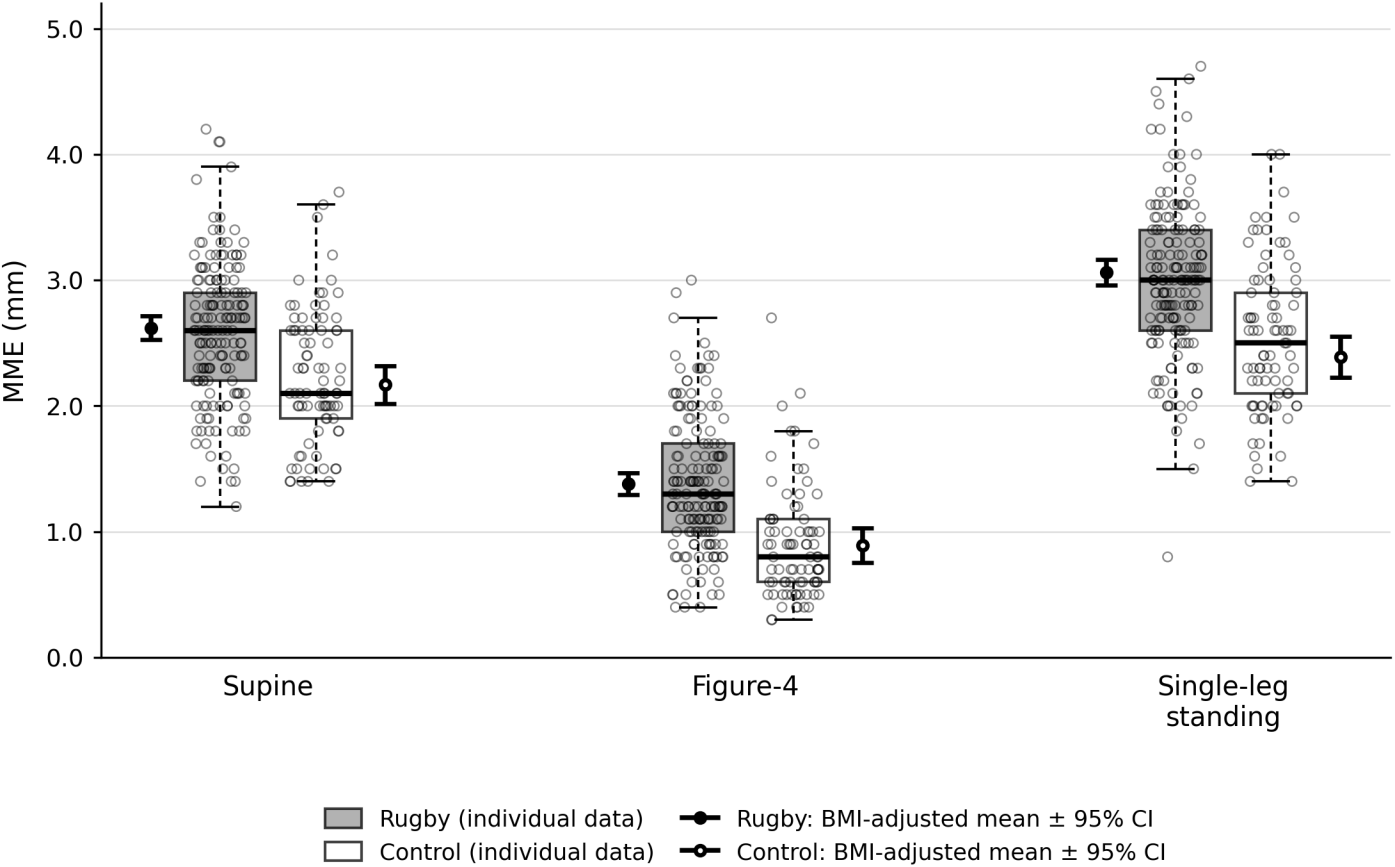
Distribution of MME values in rugby players and controls across the three evaluated knee positions. Individual points represent knee-level measurements. Box plots indicate the median, interquartile range, and range of the observed data. Filled and open circles represent BMI-adjusted estimated marginal means for rugby players and controls, respectively, derived from the linear mixed-effects models. Error bars indicate 95% CI. MME = medial meniscus extrusion. CI = confidence intervals. BMI = body mass index.

BMI-adjusted estimated marginal means of MME with 95% confidence intervals for each group across knee positions are presented in Fig 3. In the supine position, the estimated mean MME was 2.62 mm (95% CI: 2.53–2.72) in the rugby group and 2.17 mm (95% CI: 2.02–2.32) in the control group. In the figure-4, the corresponding values were 1.38 mm (95% CI: 1.29–1.47) and 0.89 mm (95% CI: 0.75–1.03), respectively. In the single-leg standing position, the estimated mean MME was 3.06 mm (95% CI: 2.96–3.17) in the rugby group and 2.39 mm (95% CI: 2.23–2.55) in the control group.

## Discussion

The present study examined whether MME is already increased in rugby players compared with controls with different levels of sport exposure, even among young individuals without a history of knee joint injury. The results demonstrated that the rugby group exhibited significantly greater MME than the control group across all measured knee positions. Previous studies have shown that MME is associated with cartilage volume loss and disease progression even in the early stages of KOA [3,6,12]. Therefore, the increased MME observed in young rugby players in the present study represents a finding suggestive of an elevated future risk of KOA. However, because this study employed a cross-sectional design, a causal relationship between rugby-related exposure and increased MME cannot be established, and longitudinal studies are required to clarify the temporal relationship between sport exposure and changes in MME.

One potential mechanism underlying the increased MME observed in the rugby group involves impairment of the hoop function of the medial meniscus due to repetitive overload or micro damage at the medial meniscus root. In the present study, MME was greater in the rugby group in both single-leg standing and the supine and figure-4 positions, indicating that the observed increase cannot be attributed solely to weight-bearing conditions and may reflect alterations in the intrinsic support mechanisms of the meniscus. Supporting this interpretation, a previous study comparing knees with medial meniscus root tears and healthy knees reported that significantly greater MME was observed in the injured knees even in the supine position [39]. In addition, figure-4 is considered a non–weight-bearing position but involves varus stress at the knee joint. A study comparing posterior root tear, degenerative tear, and healthy groups demonstrated that MME in figure-4 was significantly greater in the posterior root tear group than in the other groups [38]. However, the present study did not directly assess meniscal root integrity or cartilage morphology; therefore, these interpretations should be considered speculative. In addition, the studies cited above were conducted in populations with injured knees, and thus may not be directly comparable to clinically healthy young athletes. Further studies using advanced imaging modalities, such as MRI, are required to directly evaluate meniscal root integrity and related structural changes and to test these hypotheses.

Another potential mechanism underlying the increased MME observed in the rugby group involves the formation of micro–cartilaginous osteophytes at the proximal tibia, which can compress the coronary ligaments, including the meniscotibial ligament (MTL), thereby displacing the medial meniscus outward. The MTL, also referred to as a coronary ligament, plays a key role in anchoring the peripheral margin of the meniscus to the tibial plateau [41]. Ishijima et al. [42] reported that the degree of MME corresponded to the width of medial tibial osteophytes when the cartilaginous component was included in the assessment and emphasized that cartilaginous osteophytes—often difficult to detect on plain radiographs or conventional MRI—tend to be underestimated. Osteophyte formation is regarded as a secondary structural change or a physiological response to increased mechanical loading. Accordingly, in populations exposed to repetitive knee joint loading, such as rugby players, micro–cartilaginous osteophyte formation can plausibly progress from a young age before becoming radiographically apparent. Taken together, these findings suggest an association between repetitive loading in rugby and increased MME; however, the underlying mechanisms remain unclear, and further studies incorporating direct assessment of osteophytes, including their cartilaginous components, and consideration of body composition are warranted.

In the present study, US was used to evaluate MME. Although US is not considered the gold standard for meniscal assessment, it allows functional evaluation under biomechanically relevant conditions, which is particularly advantageous for athletes who are routinely exposed to high mechanical stress. A recent systematic review and meta-analysis evaluating the reliability of US-based MME assessment and its association with MRI findings in both KOA patients and healthy individuals demonstrated high intra- and inter-rater reliability, strong correlations with MRI measurements, and superior visualization of meniscal extrusion under weight-bearing conditions [43]. Furthermore, a recent narrative review of adult studies reported that US provides highly reproducible measurements and is particularly suitable for assessing meniscal behavior under dynamic conditions [33]. Collectively, these findings support the use of US in the present study and suggest that the measurement accuracy was not compromised relative to MRI, while offering advantages in evaluating functional meniscal changes.

An unexpected finding was the negative BMI coefficients observed in some models, despite previous studies reporting positive associations between BMI and MME [1,6,36]. A likely explanation is that rugby players exhibited markedly higher BMI values than controls and that the overlap between BMI distributions was limited. Under these conditions, the within-group association estimated by the model may differ from the overall association observed across mixed populations and may even diverge from the direction of the marginal association. Moreover, previous studies reporting positive associations between BMI and MME have primarily focused on general populations, middle-aged and older adults, or individuals with KOA, whereas evidence in highly competitive young athletes remains limited. Therefore, it remains unclear whether the BMI–MME relationship observed in these populations can be directly applied to highly competitive young athletes. Accordingly, the BMI-adjusted estimates reported in the present study should be interpreted with caution. Nevertheless, significantly greater MME values were consistently observed in rugby players across all evaluated positions, suggesting that the observed group differences are unlikely to be explained solely by BMI.

In general, MME exceeding 3 mm has been reported to be associated with the onset and progression of KOA [1,36]. For example, a systematic review including 26 studies reported that the weighted mean MME measured by US was 1.84 ± 0.67 mm in the supine position in healthy individuals, whereas it was 3.83 ± 0.65 mm in patients with KOA [40]. For reference, in patients with meniscal root tears, MME measured by US was 3.6 ± 1.0 mm in the supine position and 3.7 ± 0.9 mm under full weight-bearing conditions [39]. Furthermore, according to a study of athletes with healthy knees, although the definition of MME differed slightly from that used in the present study, participants in a mountain ultramarathon (mean age: 37.4 ± 8.3 years) showed baseline MME values of 1.9 ± 0.3 mm in the supine position and 2.4 ± 0.4 mm under full weight-bearing conditions prior to the race [44]. In the present study, MME did not exceed this threshold in most positions in either group, suggesting that the observed differences do not represent overt pathological changes. By contrast, previous finite element studies have demonstrated that each 1 mm increase in MME significantly increases mechanical loading on the meniscus and tibial cartilage [19]. Therefore, even a between-group difference of approximately 0.5 mm can reflect increased joint loading from a biomechanical perspective. Furthermore, considering that MME is known to increase with age [1,36], the finding that young rugby players exhibited greater extrusion than controls in this study may indicate early mechanical alterations in the knee joint and a potential increased risk of developing KOA in the future. However, it should be noted that the 3 mm threshold has primarily been proposed based on studies involving middle-aged or general populations, and its applicability to young athletes remains uncertain.

Several limitations of this study should be acknowledged. First, analyses stratified by playing position were not performed, although knee joint loading likely differs by position in rugby. Second, intra-rater reliability was assessed in only five participants, and all measurements were conducted by a single examiner who was not blinded to group allocation, which could limit external validity and introduce potential measurement bias. Third, prior knee injury was assessed using self-reported questionnaires, and minor or undiagnosed conditions may not have been fully captured; participants with clinically significant injuries were excluded, and all individuals were actively competing at the time of assessment. Fourth, the measurement order was fixed, and thus potential order effects cannot be completely ruled out. Fifth, although the use of a single-leg standing position reflects sport-specific loading conditions, it limits direct comparison with studies using double-leg protocols. Sixth, no a priori sample size calculation was performed, and the statistical power to detect small effects may be limited. In addition, separate models were constructed for each knee position to facilitate interpretation of between-group differences. However, this simplified modeling approach did not account for potential correlations across knee positions within participants, representing a potential limitation in the analytical strategy and interpretation of the results.

## Conclusion

In conclusion, the present study demonstrated that young rugby players without an apparent history of knee joint injury exhibited significantly greater MME than control participants across all measured knee positions. Given that MME is considered an early structural marker associated with the development of KOA, the present findings indicate that rugby players may already be at an increased risk of future KOA, even at a young age and in the absence of clinically evident knee pathology. However, causality cannot be inferred, and longitudinal studies are required to clarify the direction and underlying mechanisms of these associations.

## Supporting information

S1 File Data public anonymized. (XLSX)
